# Realizing the symmetry-protected Haldane phase in Fermi–Hubbard ladders

**DOI:** 10.1038/s41586-022-04688-z

**Published:** 2022-06-01

**Authors:** Pimonpan Sompet, Sarah Hirthe, Dominik Bourgund, Thomas Chalopin, Julian Bibo, Joannis Koepsell, Petar Bojović, Ruben Verresen, Frank Pollmann, Guillaume Salomon, Christian Gross, Timon A. Hilker, Immanuel Bloch

**Affiliations:** 1 Max-Planck-Institut für Quantenoptik, Garching, Germany; 2 Munich Center for Quantum Science and Technology, Munich, Germany; 3 Department of Physics and Materials Science, Chiang Mai University, Chiang Mai, Thailand; 4 Department of Physics, Technical University of Munich, Garching, Germany; 5 Department of Physics, Harvard University, Cambridge, MA USA; 6 Institut für Laserphysik, Universität Hamburg, Hamburg, Germany; 7 The Hamburg Centre for Ultrafast Imaging, Universität Hamburg, Hamburg, Germany; 8 Physikalisches Institut, Eberhard Karls Universität Tübingen, Tübingen, Germany; 9 Fakultät für Physik, Ludwig-Maximilians-Universität, München, Germany

**Keywords:** Topological matter, Quantum simulation

## Abstract

Topology in quantum many-body systems has profoundly changed our understanding of quantum phases of matter. The model that has played an instrumental role in elucidating these effects is the antiferromagnetic spin-1 Haldane chain^1,2^. Its ground state is a disordered state, with symmetry-protected fourfold-degenerate edge states due to fractional spin excitations. In the bulk, it is characterized by vanishing two-point spin correlations, gapped excitations and a characteristic non-local order parameter^3,4^. More recently it has been understood that the Haldane chain forms a specific example of a more general classification scheme of symmetry-protected topological phases of matter, which is based on ideas connected to quantum information and entanglement^5–7^. Here, we realize a finite-temperature version of such a topological Haldane phase with Fermi–Hubbard ladders in an ultracold-atom quantum simulator. We directly reveal both edge and bulk properties of the system through the use of single-site and particle-resolved measurements, as well as non-local correlation functions. Continuously changing the Hubbard interaction strength of the system enables us to investigate the robustness of the phase to charge (density) fluctuations far from the regime of the Heisenberg model, using a novel correlator.

## Abstract (web summary)

A ladder-like arrangement of an ultracold gas of lithium atoms trapped in an optical lattice enables the observation of a symmetry-protected topological phase.

## Main

Topological phases of matter often share a deep connection between their bulk and edge properties^8,9^. In the case of the Haldane chain, the bulk exhibits a hidden antiferromagnetic (AFM) order characterized by AFM correlations interlaced with an arbitrary number of *S*^*z*^ = 0 elements, where *S*^*z*^ denotes the spin component in the z-direction. This pattern can only be revealed through non-local string correlations that are sensitive to the local spin states, which, however, require detection of the quantum many-body system with microscopic resolution. Although this was not possible in early experiments on spin-1 chains, evidence for a spin gap, as well as spin-1/2 localized edge states, was found using neutron scattering^10,11^ or electron resonance experiments^12,13^ while not directly probing this hidden order or spatially resolving the edge states. Recent developments in quantum simulations enable one to go beyond such solid-state bulk measurements by observing quantum many-body systems with single-site resolution^14–18^ and in a fully spin- and density-resolved way^19,20^. This provides a rich diagnostic tool to obtain a direct microscopic picture of the hidden order in experiments^21,22^. The power of this technique has also been demonstrated recently in a study that was able to reveal a symmetry-protected topological (SPT) phase in the hardcore boson Su–Schrieffer–Heeger (SSH) model using Rydberg atoms^23^. Here we expand on those results by realizing a finite-temperature version of the Haldane phase in a spin system with tuneable coupling strength, system size and controlled charge fluctuations. We show this by measuring both topological and trivial string order parameters.

An instructive way to engineer the Haldane phase in systems of spin-1/2 fermions is on the basis of the AKLT model^4,24^, in which a spin-1 particle is formed out of two spin-1/2 particles. Thus, spin-1/2 ladder systems emerge as an experimentally realizable platform for the Haldane phase. Whereas a natural implementation with spin-1 particles on individual rungs requires ferromagnetic rung couplings and antiferromagnetic leg couplings, a much wider variety of couplings in spin-1/2 quantum ladders feature the Haldane phase^25,26^. This includes the antiferromagnetic Heisenberg case, which we realize here as the strong-interaction limit of the Fermi–Hubbard model.

**Fig. 1:**
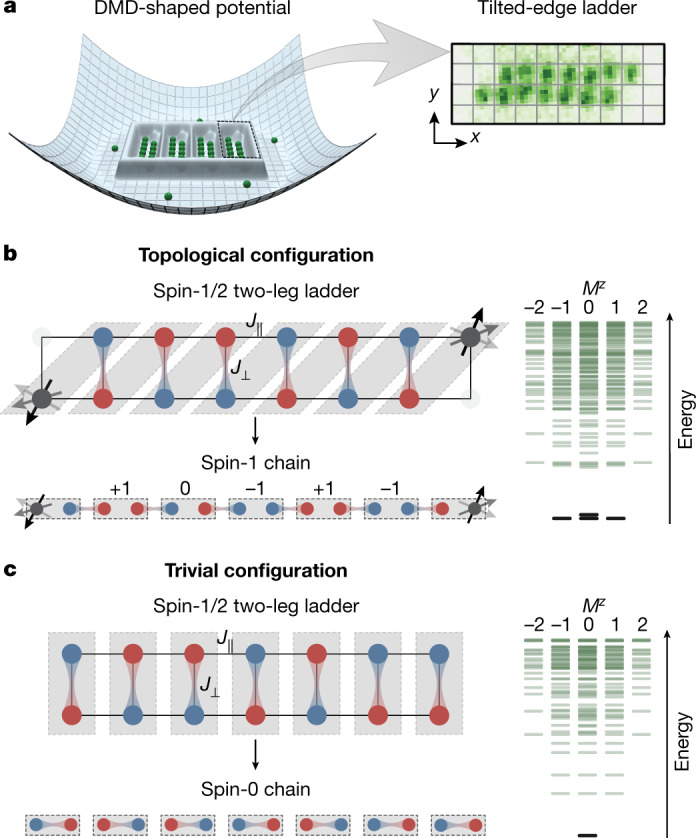
Probing topological phases in spin-1/2 ladders of cold atoms. **a**, Realization of tailored spin-1/2 ladders in a single plane of a 3D optical lattice with a potential shaped by a DMD. The dilute wings of the potential are well separated from the homogeneous ladder systems. Using quantum gas microscopy, we obtain fully spin- and density-resolved images of the system. The inset shows a single-shot fluorescence image of the prepared ladder without spin resolution. **b**, **c**, Connecting spin-1/2 ladders to integer-spin chains by grouping pairs of spins in unit cells. For diagonal unit cells (**b**) the AFM Heisenberg ladder adiabatically connects to the Haldane spin-1 chain showing spin-1/2 edge states and hidden long-range order (that is, AFM order interspersed with *S*^*z*^ = 0 unit cells). For vertical unit cells (**c**), the system is in the topologically trivial phase dominated by singlets on the rungs, forming a spin-0 chain. We adapt the edges of the system to match the respective unit cell, that is straight edges for vertical unit cells and tilted edges for diagonal unit cells, which we realize by blocking one site on each end. The energy spectra of the systems grouped by total magnetization *M*^*z*^ display gapped fourfold near-degenerate ground states for the topological configuration and a single ground state for the trivial one. Sketch for *L* = 7.

In our experiment, we prepare such ladders by adiabatically loading a spin-balanced mixture of the two lowest hyperfine states of ^6^Li into an engineered lattice potential (Methods). As illustrated in Fig. 1a, we realize four isolated two-leg ladders with a variable number (*L*) of unit cells (where *L* is therefore also equivalent to length), surrounded by a low-density bath of particles^27^. The unit cells are chosen to be either along the rungs of the ladders (vertical unit cell, Fig. 1b) or along the diagonals (diagonal unit cells, Fig. 1c). The edges of the ladders are then engineered to match the choice of unit cell: straight edges are chosen for vertical unit cells, whereas one site is blocked on each edge in the case of diagonal unit cells. The atoms in the lowest band of the optical lattice are well described by the Fermi–Hubbard model, with tunnelling energies, ${t}_{\parallel }$ (chain), ${t}_{\perp }$ (rung), and on-site interactions *U*. For half-filling and at strong $U/{t}_{\parallel ,\perp }\approx 13$, used throughout most of our experiments (see Methods for details), density fluctuations are suppressed and the spin ladder realizes the Heisenberg model^28^ with Hamiltonian:

1 $$\hat{H}={J}_{\parallel }\sum _{\begin{array}{c}x\in [0,L)\\ y=A,B\end{array}}{\hat{{\bf{S}}}}_{x,y}\cdot {\hat{{\bf{S}}}}_{x+1,y}+{J}_{\perp }\sum _{x\in [0,L)}{\hat{{\bf{S}}}}_{x,A}\cdot {\hat{{\bf{S}}}}_{x,B}$$

with positive leg and rung couplings, ${J}_{\parallel ,\perp }=4{t}_{\parallel ,\perp }^{2}/U$ and the spin-1/2 operators ${\hat{{\bf{S}}}}_{x,y}$ at site (*x*, *y*), with *A*, *B* denoting the two legs of the ladder.

The topological properties are most easily explained in the limit ${J}_{\perp }\gg {J}_{\parallel }$, where strong spin singlets form along the rungs and the system exhibits an energy gap of ${J}_{\perp }$. The behaviour on the edges of the ladder then depends on how the system is terminated, and therefore on which unit cells have been chosen. For diagonal unit cells (Fig. 1b), two unpaired spin-1/2 particles remain and the many-body system has a fourfold degeneracy that is only weakly lifted by an edge-to-edge coupling, which vanishes exponentially with system size (Supplementary Information). In the trivial case of vertical unit cells (Fig. 1c), all spins pair into singlets and the ground state is unique. These descriptions remain valid even for weaker ${J}_{\perp }/{J}_{\parallel }$, where the singlet alignment may change between vertical and horizontal, but any line between two rungs cuts an even number of singlets^29,30^.

To make the analogy between the spin-1/2 ladder and the Haldane integer chain more apparent, we switch to a description in terms of total spin per *k*th unit cell, ${\hat{{\bf{S}}}}_{k}={\hat{{\bf{S}}}}_{k,{\rm{A}}}+{\hat{{\bf{S}}}}_{k,{\rm{B}}}$, where the indices (A, B) indicate the two spin-1/2 particles in the same unit cell, making an integer spin. In the diagonal unit cell such a system shows a high (≥80%) triplet fraction^26^ (Supplementary Information). We note that this spin ladder can be adiabatically connected to a spin-1 chain by including ferromagnetic couplings within the unit cell^25^. However, having a high triplet fraction is not essential for having a well-defined Haldane phase, as both systems share the same universal SPT features^26^.

**Fig. 2:**
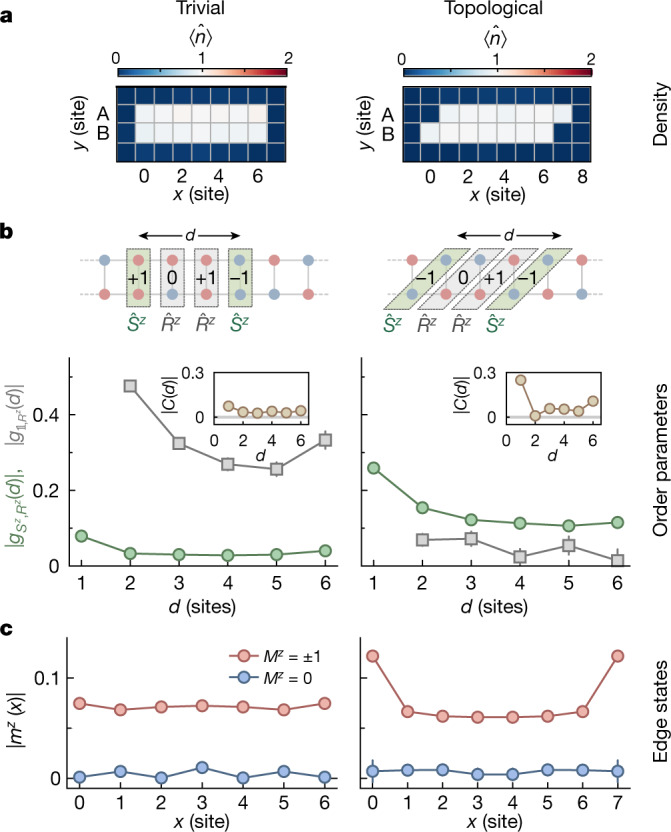
Trivial versus topological configurations. **a**, The atomic density distribution ⟨$\hat{n}$⟩ of ladders with diagonal and vertical unit cells. **b**, The amplitudes of the spin-string correlator ${g}_{{S}^{z},{R}^{z}}$ (green circles) and the string-only correlator ${g}_{{\mathbb{1}},{R}^{z}}$ (grey squares) observed as a function of the spin distance over *d* unit cells. The cartoon illustrates the unit cells, the total spin *S*^*z*^ per unit cell and the string correlators for a subsystem with *d* = 3. In the trivial configuration (rung unit cells), $|{g}_{{\mathbb{1}},{R}^{z}}(d)|$ is well above zero, whereas $|\,{g}_{{S}^{z},{R}^{z}}(d)|$ is rapidly vanishing at *d* > 1. By contrast, for the topological configuration (diagonal unit cells), $|\,{g}_{{S}^{z},{R}^{z}}(d)|$, shows a long-range correlation, whereas $|{g}_{{\mathbb{1}},{R}^{z}}(d)|$ is close to zero. In both cases, the two-point spin–spin correlation *C*(*d*) decays rapidly to zero as a function of the distance *d* (insets). The correlators ${g}_{{\mathbb{1}},{R}^{z}},{g}_{{S}^{z},{R}^{z}}$ and *C*(*d*) are evaluated for fixed total magnetization ${m}^{z}$  = 0. **c**, Amplitudes of the rung- and inversion-averaged local magnetizations $|{m}^{z}(x)|$ plotted as a function of position *x* along the chains for different ${m}^{z}$. In the unbalanced spin sector of the topological configuration (${m}^{z}$ = ±1), the result displays a localization of the excess spins at the edges, signalling the presence of edge states. All data were taken with ${J}_{\perp }/{J}_{\parallel }=1.3(2)$. Error bars denote one standard error of the mean (s.e.m.) and are smaller than their marker size if not visible. Source data

The defining property of the Haldane SPT phase is that it is an integer-spin chain with spin-1/2 edge modes: the bulk SO(3) symmetry is said to fractionalize into SU(2) symmetry at the edge. It has no spontaneous symmetry breaking and thus the spin correlation function $\langle {\hat{S}}_{k}^{z}{\hat{S}}_{k+d}^{z}\rangle$ is short range. Instead, the aforementioned symmetry fractionalization^6,7^ can be detected in the bulk using string order parameters^3,31^:

2 $${g}_{{\mathscr{O}},U}(d)=\langle {\hat{{\mathscr{O}}}}_{k}(\mathop{\prod }\limits_{l=k+1}^{k+d-1}{\hat{U}}_{l}){\hat{{\mathscr{O}}}}_{k+d}\rangle $$

with an on-site symmetry ${\hat{U}}_{l}$ and endpoint operator ${\hat{{\mathscr{O}}}}_{k}$, where *l* denotes the unit cell and *d* the string distance (Fig. 2 and Supplementary Information). This correlator probes the transformation behaviour of the bulk under a symmetry ${\hat{U}}_{l}$; for example, a spin rotation around the *z* axis by π, ${\hat{R}}_{l}^{z}\equiv {\rm{\exp }}({i}{\rm{\pi }}{\hat{S}}_{l}^{z})$. The pure-string correlator ${g}_{{\mathbb{1}},{R}^{z}}(d)$, where ${\hat{{\mathscr{O}}}}_{k}=1$ and ${\hat{U}}_{l}={\hat{R}}_{l}^{z}$, is non-zero for $d\gg 1$ if the edge does not have half-integer spins^31^. This is the case for the topologically trivial configuration but not for the Haldane phase, in which the symmetry is fractionalized. The spin-string operator ${g}_{{S}^{z},{R}^{z}}(d)$(ref. ^3^), ${\hat{{\mathscr{O}}}}_{k}={\hat{S}}_{k}^{z}$, exhibits the opposite behaviour and is non-zero only in the Haldane phase (see Supplementary Information for details about the symmetries of the Haldane phase). Thus we can identify the Haldane phase by comparing the two string correlators ${g}_{{S}^{z},{R}^{z}}$ and ${g}_{{\mathbb{1}},{R}^{z}}$, and observe opposite behaviour in the two different regimes.

Another perspective on ${g}_{{S}^{z},{R}^{z}}$ can be gained by recognizing it as a normal two-point correlator at distance *d*, which ignores all spin-0 contributions along the way (‘squeezed space’^22,32^). In the underlying spin-1/2 system, this order stems from *N *− 1 consecutive rungs dominantly consisting of *N *− 1 singlets and two spin-1/2 states, which have a combined total spin of +1, 0 or −1.

To observe the characteristics of the SPT phase, we prepare a two-leg ladder of length *L* = 7 and ${J}_{\perp }/{J}_{\parallel }=1.3(2)$ in both the topological and the trivial configuration. The tailored potential yields a homogeneous filling of the system with sharp boundaries (Fig. 2a), which is characterized by a remaining density variance over the system of 2 × 10^−4^. To focus on the spin physics, we select realizations with ${N}_{\uparrow }+{N}_{\downarrow }=2L$ per ladder. Additionally, we exclude ladders with an excessive number of doublon–hole fluctuations and do not consider strings with odd atom numbers in the string or the endpoints of the correlator (Methods). We characterize the spin-balanced ladder systems $({M}^{z}\equiv ({N}_{\uparrow }-{N}_{\downarrow })/2=0)$ by evaluation of the string order parameters, as defined in equation (2). In the topological configuration, we observe fast decay of ${g}_{{S}^{z},{R}^{z}}$ over a distance of approximately one site and a long-range correlation up to *d* = 6, with a final value of ${g}_{{S}^{z},{R}^{z}}\simeq 0.1$ (Fig. 2b). In contrast, for the trivial configuration, the correlation decays rapidly to zero as a function of the string correlator length. The opposite behaviour is seen for ${g}_{{\mathbb{1}},{R}^{z}}(d)$, demonstrating the hidden correlations expected for both phases.

Furthermore, the two-point spin correlation, $$C(d)\equiv {g}_{{S}^{z},{\mathbb{1}}}(d)=$$$$\langle {\hat{S}}_{k}^{z}{\hat{S}}_{k+d}^{z}\rangle \,,$$ yields only the short-range AFM correlation characteristic for a gapped phase (see insets in Fig. 2b). It is interesting to note that at the largest distance in the topological case, *C*(*d* = 6) displays a clear (negative) correlation between the two edge spins, despite small correlations at shorter distances. This (classical) correlation confirms the existence of a non-magnetized bulk, such that spins on the edges of the system must be of opposite direction at global *M*^*z*^ = 0.

We probe the edges explicitly by measuring the amplitude of the local rung-averaged magnetization *m*^*z*^(*x*) as a function of rung position *x* for different sectors of the ladder magnetization *M*^*z*^ (Fig. 2c). In the case of an imbalanced spin mixture with *M*^*z*^ = ±1, we see that the two end sites exhibit on average a higher magnetization than the bulk rungs in the topological configuration. This is consistent with the bulk of the ground states of both phases forming a global singlet, and only the edges of the topological phase carrying an excess spin-1/2 without energy cost. The measured bulk magnetization can be attributed to finite-temperature effects (Supplementary Information).

**Fig. 3:**
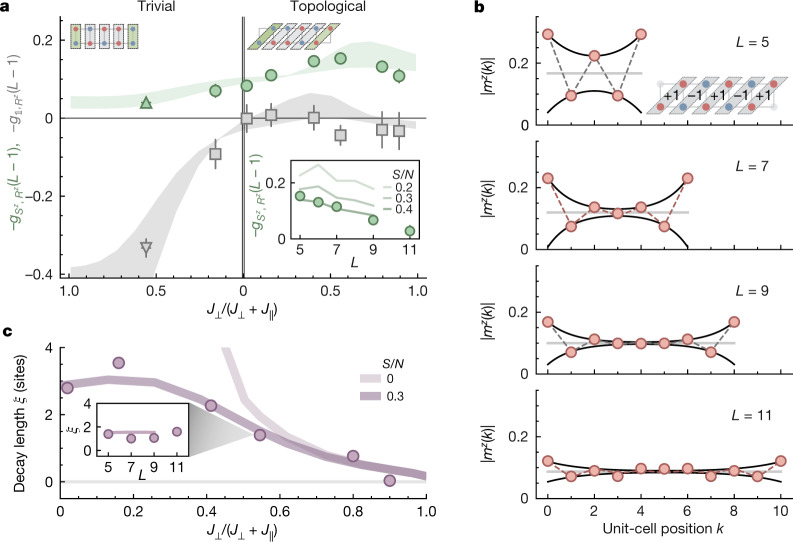
Influence of spin-coupling strength on the string order parameters and the edge states. **a**, The two string order parameters, ${g}_{{S}^{z},{R}^{z}}$ (green) and ${g}_{{\mathbb{1}},{R}^{z}}$ (grey), for both trivial (left) and topological (right) configurations measured as a function of ${J}_{\perp }/({J}_{\perp }+{J}_{\parallel })$. Both ${g}_{{S}^{z},{R}^{z}}$ and ${g}_{{\mathbb{1}},{R}^{z}}$ stay finite in their respective phases and are largely consistent with zero in the other phase. The data were taken at a chain length of *L* = 5 except for one data point marked by a triangle at *L* = 7. Shaded curves are the ED results of the two order parameters at finite entropy per particle, *S*/*N* = (0.3−0.45) *k*_B_ and *L* = 5. The inset shows the measured ${g}_{{S}^{z},{R}^{z}}$ as a function of the chain length *L* at ${J}_{\perp }/{J}_{\parallel }=1.3(2)$ (that is, ${J}_{\perp }/({J}_{\perp }+{J}_{\parallel })=0.56(4)$). The decay in the magnitude of the string order parameter with length is expected at finite temperatures in quantitative agreement with ED results (lines) at *S*/*N* ≈ 0.4 *k*_B_. **b**, Edge state localization at ${J}_{\perp }/{J}_{\parallel }=1.3(2)$. In the ${m}^{z}$ = ±1 spin sectors of the topological configuration, the unit cell local magnetization $|{m}^{z}(k)|$ at chain position *k* shows excess magnetization localized at the edges for different lengths. The black line is a fit to our inversion-averaged data. **c**, The localization length *ξ* of the edge states increases with the leg coupling ${J}_{\parallel }$ but saturates at a value set by temperature and system size *L* = 5. Lines are ED results at *S*/*N* = 0.3 *k*_B_ and 0 *k*_B_. The inset shows the independence of *ξ* with respect to *L* extracted from the plots in **b**, as well as ED results for *S*/*N* = 0.3 *k*_B_. Error bars denote one standard error of the mean (s.e.m) and are smaller than their marker size if not visible. Source data

The SPT phase is expected to be robust^26^ on variation of the ratio ${J}_{\perp }/{J}_{\parallel }$, but maintains a finite gap in the system. We realize both the trivial and topological configuration with different ${t}_{\perp }/{t}_{\parallel }$ at almost fixed *U* and study the string correlators at maximal distance (*L *− 1) versus ${J}_{\perp }/{J}_{\parallel }$ (Fig. 3a). For the topological configuration with diagonal unit cells, we observe ${g}_{{\mathbb{1}},{R}^{z}}(L-1)\simeq 0$ and $|{g}_{{S}^{z},{R}^{z}}| > 0$ for all ${J}_{\perp }/{J}_{\parallel }$ with a maximum around ${J}_{\perp }/{J}_{\parallel }\simeq 1.3(2)$ (that is, ${J}_{\perp }/({J}_{\perp }+{J}_{\parallel })\simeq 0.56(4)$), whereas for the trivial case the role of the correlators is reversed. Both phases continuously connect in the limit of two disconnected chains at ${J}_{\perp }=0$. These observations demonstrate qualitatively all the key predictions of the antiferromagnetic spin-1/2 ladder at temperature *T* = 0 (ref. ^26^) and the strengths of the measured correlations are consistent with exact diagonalization (ED) calculations using an entropy per particle $S/N=(0.3-0.45)\,{k}_{{\rm{B}}}$ (shaded lines in Fig. 3a).

We reveal these features despite the finite temperature in our system, which would destroy the long-range hidden order in an infinite system. The total entropy in our system is, however, still low enough to yield a large fraction of realizations of the topological ground state. In larger systems, the total number of thermal excitations grows (at fixed entropy per particle) and the non-local correlator $|\,{g}_{{S}^{z},{R}^{z}}(L-1)|$ decreases (see inset of Fig. 3a), consistent with vanishing correlations in the thermodynamic limit, thus yielding a restriction on our system size at our level of experimental precision and entropy per particle (Supplementary Information). Finite size effects are explored in detail in the Supplementary Information. We note that the difference between the SPT phase and the trivial phase is here clearly shown by considering both ${g}_{{S}^{z},{R}^{z}}$ and ${g}_{{\mathbb{1}},{R}^{z}}$.

To investigate the localization length of the edge states, we evaluate our data for ${m}^{z}$ = ±1 and plot the local magnetization per unit cell ${m}^{z}$(*k*) for different system sizes (Fig. 3b). Because of the singlets in the bulk, the excess spin is most likely to be found at the edges of the system. This spin partly polarizes the neighbouring sites antiferromagnetically, leading to an exponentially localized net magnetization with AFM substructure^33^. The data are well described by the fit function ${m}^{z}(k)={m}_{{\rm{B}}}+{m}_{{\rm{E}}}\left({(-1)}^{k}{{\rm{e}}}^{-k/\xi }+{(-1)}^{L-k-1}{{\rm{e}}}^{-(L-k-1)/\xi }\right)$ with free bulk magnetization *m*_B_, edge magnetization *m*_E_ and decay length *ξ*. In Fig. 3c, we show how this localization length *ξ* decreases as we approach the limit of rung singlets, ${J}_{\perp }\gg {J}_{\parallel }$. Comparison with ED lets us identify two regimes: at ${J}_{\perp }\gtrsim {J}_{\parallel }$, the measured *ξ* drops with larger ${J}_{\perp }$ and coincides with theory independent of temperature, whereas at low ${J}_{\parallel }$ thermal effects dominate, limiting the increase of *ξ* to three sites for our system (Supplementary Information).

Thus far, we have worked in the Mott limit where density fluctuations can be ignored, such that the spin Hamiltonian, equation (1), is a good effective description of the Fermi–Hubbard ladder. However, it is known that the Haldane SPT phase can be unstable to density fluctuations^34–36^. By reducing $U/{t}_{\parallel }$, the symmetry in the unit cell in the bulk changes from SO(3) to SU(2), as it now may contain both half-integer and integer total spin. This effectively removes the distinction between bulk and edge (Supplementary Information). This means that the edge mode and string order parameter are no longer topologically non-trivial, which is also manifested in the fact that the two phases can be adiabatically connected by tuning through a low-$U/{t}_{\parallel }$ regime if one breaks additional symmetries but preserves spin-rotation symmetry^34–36^. In particular, the above string orders lose their distinguishing power: ${g}_{{S}^{z},{R}^{z}}$ and ${g}_{{\mathbb{1}},{R}^{z}}$ will both generically have long-range order away from the Mott limit^34^.

Intriguingly, despite the breakdown of the above symmetry argument and string order parameter, the Hubbard ladder (with diagonal unit cell) remains a non-trivial SPT phase due to its sublattice symmetry. This symmetry is a direct consequence of the ladder being bipartite (see Supplementary Information for details). It is simplest to see that this protects the SPT phase in the limit *U* = 0, where the two spin species decouple, such that our model reduces to two copies of the SSH chain^37^. It is known that such a stack remains in a non-trivial SPT phase in the presence of interactions, namely *U* ≠ 0 (ref. ^38^). Moreover, together with the parity symmetry of spin-down particles, ${\hat{P}}_{l}^{\downarrow }\equiv {\rm{\exp }}\left[{i}{\rm{\pi }}\left({\hat{n}}_{l,{\rm{A}}}^{\downarrow }+{\hat{n}}_{l,{\rm{B}}}^{\downarrow }\right)\right]$, it then gives rise to a different string order parameter: the topological phase is characterized by long-range order in ${g}_{{S}^{z},{P}^{\downarrow }}$, whereas it has vanishing correlations for ${g}_{{\mathbb{1}},{P}^{\downarrow }}$, with the roles being reversed in the trivial phase. This novel string order is derived in the Supplementary Information. Remarkably, in the Heisenberg limit, it coincides with the conventional string order parameter used before.

**Fig. 4:**
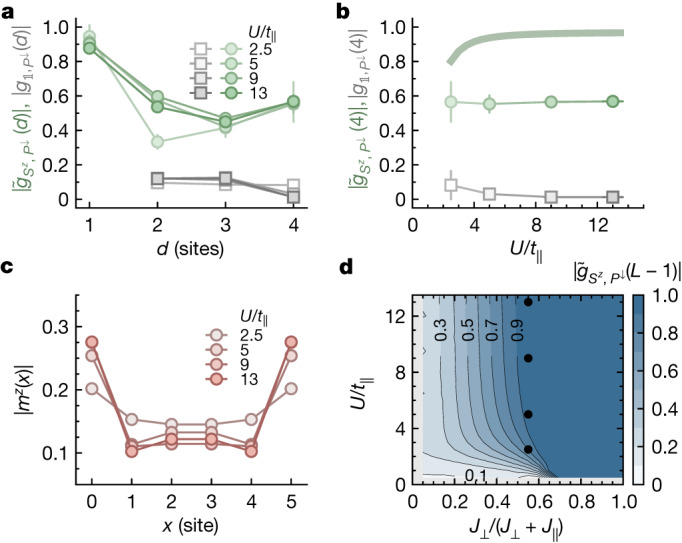
Robustness of the Haldane phase to density fluctuations. **a**, **b**, The hidden SPT order is preserved even at low Hubbard interactions, as revealed by the novel string correlators $|\,{\tilde{g}}_{{S}^{z},{P}^{\downarrow }}(d)\,|$ (green circles) and $|{g}_{{\mathbb{1}},{P}^{\downarrow }}(d)|$ (grey squares) on the basis of the spin-down parity ${\hat{P}}^{\downarrow }$. $|\,{\tilde{g}}_{{S}^{z},{P}^{\downarrow }}\,|$ stays non-zero, whereas $|{g}_{{\mathbb{1}},{P}^{\downarrow }}|$ is consistent with zero for *d* = *L *− 1 over the measured interaction range. The same qualitative behaviour is seen in zero-temperature DMRG calculations (shaded line) with $L\to \infty$. **c**, Spatial distribution of excess magnetization (${m}^{z}$ = ±1) for decreasing $U/{t}_{\parallel }$. Even far away from the Heisenberg regime, the edge state signal remains strong and only diminishes for very weak $U/{t}_{\parallel }$. **d**, Map of zero-temperature DMRG $(L\to \infty )$ results for the spin-string correlator in the entire parameter space of the topological phase. It shows a strictly non-zero ${\hat{g}}_{{S}^{z},{P}^{\downarrow }}$ while ${g}_{{\mathbb{1}},{P}^{\downarrow }}(L-1)=0$ everywhere in this phase. The black circles indicate the parameters of the measurements. All experimental data were taken at ${J}_{\perp }/{J}_{\parallel }=1.3(2)$ and *L* = 5 in the tilted geometry. ${m}^{z}$ = 0 in **a**, **b** and **d**. Error bars denote one standard error of the mean (s.e.m.) and are smaller than their marker size if not visible. Source data

In the topological phase it is meaningful to normalize ${g}_{{S}^{z},{P}^{\downarrow }}$ to ${\tilde{g}}_{{S}^{z},{P}^{\downarrow }}=\eta {g}_{{S}^{z},{P}^{\downarrow }}$ with ${\eta }^{-1}=\langle |{\hat{S}}_{k}^{z}||{\hat{S}}_{k+d}^{z}|\rangle$, which effectively excludes endpoints with spin *S*^*z*^ = 0. Indeed, we find unchanged string correlations ${\tilde{g}}_{{S}^{z},{P}^{\downarrow }}$ and ${g}_{{\mathbb{1}},{P}^{\downarrow }}$ down to the lowest experimentally explored value $U/{t}_{\parallel }=2.5(2)$ (Fig. 4a, b) and edge state signals down to $U/{t}_{\parallel }=5.0(3)$ (Fig. 4c). Density matrix renormalization group (DMRG) calculations for $L\to \infty$ confirm non-zero ${\tilde{g}}_{{S}^{z},{P}^{\downarrow }}$ (*L*−1) at *T* = 0 and for all rung-coupling strengths (Fig. 4d), while ${g}_{{\mathbb{1}},{P}^{\downarrow }}(L-1)$ is strictly zero. Owing to the normalization ${\tilde{g}}_{{S}^{z},{P}^{\downarrow }}(L-1)$ goes to 1 for ${J}_{\perp }\gg {J}_{\parallel }$.

In our work, we realized a finite-temperature version of the topological Haldane SPT phase using the full spin and density resolution of our Fermi quantum gas microscope. We demonstrated the robustness of the edge states and the hidden order of this SPT phase in both the Heisenberg and the Fermi–Hubbard regime. In the future, studies may extend the two-leg ladder to a varying number of legs, in which one would expect clear differences between even and odd numbers of legs^39^ and topological effects away from half-filling^40^, or may investigate topological phases in higher dimensions^41^. Furthermore, the ladder geometry holds the potential to reveal hole–hole pairing^42^ at temperatures more favourable than in a full two-dimensional system.

## Methods

### Experimental sequence

In each experimental run, we prepare a cold atomic cloud of ^6^Li in a balanced mixture of the lowest two hyperfine states (*F* = 1/2, *m*_*F*_ = ±1/2). For evaporation, we confine the cloud in a single layer of a staggered optical superlattice along the *z* direction, with spacings *a*_s_ = 3 μm and *a*_1_ = 6 μm and depths ${V}_{s}=45\,{E}_{{\rm{R}}}^{{\rm{s}}}$ and ${V}_{l}=110\,{E}_{{\rm{R}}}^{{\rm{l}}}$, where *E*_R_ denotes the recoil energy of the respective lattice. The atoms are harmonically confined in the *xy* plane and the evaporation is performed by ramping up a magnetic gradient along the *y* direction^20^. The final atom number is tuned via the evaporation parameters.

The cloud is then loaded into an optical lattice in the *xy* plane with spacings *a*_*x*_ = 1.18 μm and *a*_*y*_ = 1.15 μm, which is ramped up within 100 ms to its final value, ranging from 5*E*_R_ to 15*E*_R_ depending on the chosen Hubbard parameters. The scattering length is tuned from 230 *a*_B_ during evaporation, with *a*_B_ being the Bohr radius, to its final value ranging between 241 *a*_B_ and 1,200 *a*_B_ using the broad Feshbach resonance of ^6^Li. An overview of the parameters of each dataset is given in Extended Data Table 1. Simultaneously with the lattice loading, a repulsive potential is ramped up, which compensates for the harmonic confinement generated by the curvature of the Gaussian lattice beams and divides the resulting flat area into four disconnected ladder systems along the *y* direction (see ‘Potential shaping’). We achieve temperatures of ${k}_{{\rm{B}}}T\approx 0.9(3){J}_{\parallel }$ for the parameters in Fig. 2.

For detection, the configuration is frozen by ramping the *xy* lattices to $43\,{E}_{{\rm{R}}}^{xy}$ within 250 μs. A Stern–Gerlach sequence separates the two spin species into two neighbouring planes of the vertical superlattice, which are then separated to a distance of 21 μm using the charge pumping technique described in ref. ^20^. Finally, simultaneous fluorescence images of the two planes are taken using Raman sideband cooling in our dedicated pinning lattice^43^, with an imaging time of 2.5 s. The fluorescence of both planes is collected through the same high-resolution objective. The light is then split into two paths using a polarizing beam splitter. One of the beams passes through a variable 1:1 telescope before both paths are recombined on a second polarizing beam splitter with a small spatial offset. This enables us to image both planes in a single exposure, with each plane in focus on a separate fixed position of our camera. We calibrated the relative position on the camera of the two imaged planes using 300 shots of a spin-split Mott insulator and the matching algorithm described in the supplement of ref. ^20^. The overall detection fidelity per atom was 96(1)%.

### Potential shaping

**Extended Data Fig. 1:**
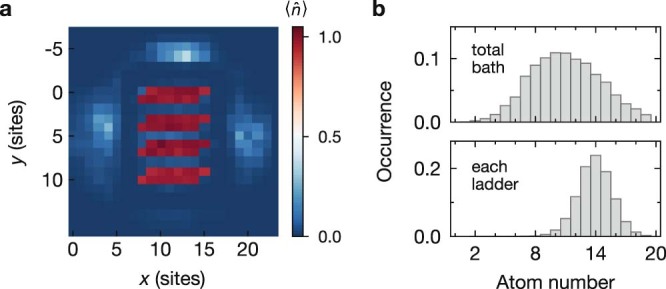
Density engineering. **a**, Repulsive light shaped with a DMD splits the system into four independent ladders in the centre surrounded by a low-density bath. The density of the ladders is *n* = 0.992 with a standard deviation of 0.03. **b**, The occupation histograms show the normalized occurrence of total atom numbers in each ladder and the normalized occurrence in the surrounding bath for *L* = 7. Almost 25% of the ladder realizations have *N* = 2*L*.

The ladder systems are created by superimposing the optical lattice with a repulsive potential, which is shaped by projecting incoherent light at 650 nm (generated from a SLED by Exalos EXS210030-03) from a digital micromirror device (DMD) through the high-resolution objective. Four ladders are created by blocking lattice sites with a potential *V* = 3.5(5)*U* around each ladder. The area outside the walled ladders is lifted above the inner ladder potential, but remains below the interaction energy *U*. It thus serves as a reservoir for surplus atoms, which occupy this region once the lowest Hubbard band of the ladders is filled. The flatness of the potential is adjusted for each parameter setting, as the intensity of the lattice beams directly influence the curvature of the potential. This is accomplished by realizing a system with about 20% doping and returning the average density of 100–150 experimental runs as feedback to the DMD pattern. We repeat the feedback until we reach a sufficiently flat density profile with a variance <1 × 10^−3^ over the 8*L* ladder sites. To adjust for drifts in the lattice phase, we continuously track the lattice phase of each experimental run and feedback to the potential position accordingly. In Extended Data Fig. 1, the average density and occupation histograms of all four ladders and the reservoir area are shown for the dataset of *L* = 7.

### Data selection

In each experimental run, four ladder systems are realized. To fulfil the criteria of the Heisenberg regime, we then select on ladder instances with atom number *N* = 2*L* and restrict the total magnetization to ${M}^{z}$ = 0, $|{M}^{z}|=1$, or $|{M}^{z}|\le 1$, depending on the observable, and specify the magnetization sector whenever data points are presented. $|{M}^{z}|\le 1$ for 87.5% of all data. Additionally, for all measurements in the Heisenberg regime, we remove ladders with more than two doublons, as those indicate a mismatch of the DMD pattern relative to the lattice phase. To give a specific example, we here give the precise numbers for the data presented in Fig. 2. This dataset consists of 7,032 realizations with four ladders each. Out of those 28,128 ladders, 6,721 have an atom number of 14. In addition, 2,636 ladders then have a total magnetization ${m}^{z}$ = 0 and 3,094 have a magnetization of ${M}^{z}$ = ± 1. Finally 77 of those 2,636 ladders have more than two doublon–hole pairs, which we exclude as these are most likely to be caused by drifts of the potential pattern given by the DMD. This leaves a total of 2,559 ladders out of the initial 28,128 for calculation of the string correlator.

For calculating the string correlators ${g}_{{S}^{z},{R}^{z}}$ and ${g}_{{\mathbb{1}},{R}^{z}}$ at fixed *d*, we exclude realizations with an odd atom number in the bulk area (grey area in the cartoon of Fig. 2b), as those would lead to imaginary contributions to the correlators, and we also exclude odd atom numbers at the edge areas (green in the cartoon of Fig. 2b). These cases are mostly due to the finite $U/{t}_{\parallel }$, which still allows for some particle fluctuations. We keep other particle–hole fluctuations, such as those occurring within the string. These do not alter the observed string correlation relative to the Heisenberg model.

### Nearest-neighbour spin correlations

**Extended Data Fig. 2:**
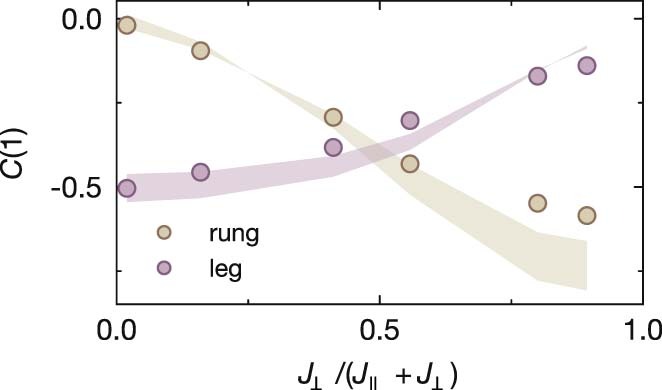
Nearest-neighbour spin correlations. The nearest-neighbour spin correlation *C*(1) for different ${J}_{\perp }/{J}_{\parallel }$ in the *L* = 5 system. The brown (purple) points refer to the correlations along the rung (leg). The shaded areas correspond to the correlations in the Heisenberg model with an entropy of *S*/*N* = (0.3−0.4) *k*_B_ per particle. Both theoretical and experimental values are obtained from the magnetization sector ${m}^{z}$ = 0.

The two-leg ladder systems show strong antiferromagnetic spin correlations in which the dominant orientation depends on the ratio of couplings ${J}_{\perp }/{J}_{\parallel }$ and the strength is measured by ${C}_{x}(d)=4\langle {\hat{S}}_{i,j}^{z}{\hat{S}}_{i,j+d}^{z}\rangle$ and ${C}_{y}=4\left\langle {\hat{S}}_{{\rm{A}},j}^{z}{\hat{S}}_{{\rm{B}},j}^{z}\right\rangle$. For a leg coupling ${J}_{\parallel }$ much higher than the rung coupling ${J}_{\perp }$, the nearest-neighbour spin correlator *C*_*y*_ along the rung almost vanishes, whereas correlations reach *C*_*x*_(1) = −0.500(6) along the leg direction. For a dominating rung coupling ${J}_{\perp }$, *C*_*y*_ reaches −0.58(1), indicating singlet formation between the two sites of a rung. Extended Data Fig. 2a shows the measured spin correlations along both rung and leg for different values of ${J}_{\perp }/{J}_{\parallel }$. The values match the finite-temperature Heisenberg model for an entropy of *S*/*N* = (0.3−0.4) *k*_B_ per particle obtained from ED.

### Theory simulation

In this work, we have used two different numerical methods to obtain theoretical predictions for the experimentally measured observables. The results in the Heisenberg regime were obtained using ED of our spin-1/2 ladders up to sizes of *L* = 9 (limited by computational resources). For each data point, the system size and geometry in the ED simulation are the same as in the experimental data. The finite-temperature results were obtained by using the full spectrum. We specify the entropy per particle *s* = *S*/*N*, which we find to be approximately independent of coupling parameters in the experimental realizations. The results in the Hubbard regime are calculated using the DMRG ansatz^44^ based on the TeNPy library (v.0.3.0)^45^. For all calculations, we conserved the total particle number and the total magnetization. For the phase diagram in Fig. 4d we used the iDMRG technique to obtain the ground state and the values of the string order parameters in the thermodynamic limit. For this, we evaluated the ground state for each parameter and used a maximal MPS bond dimension *χ* = 1,200. The bond dimension is increased in steps Δ*χ* = 40 and the simulation stopped when the difference in the ground state energy per unit cell *E*(*χ *+ Δ*χ*) − *E*(*χ*) < 10^−7^. This worked for most parameters except in the vicinity of two decoupled Hubbard chains and at small values of $U/{t}_{\parallel }$. Nevertheless, in this regime we find that the energy per unit cell is converged up to *E*(1,200) − *E*(1,160) < 10^−4^. For the experimentally accessible regime all calculations fulfil the former bound. To obtain the infinite length value of the string order parameters, we calculated it for different lengths $d\in$[200, 400, ..., 1600] to make sure that its final value is converged.

## Online content

Any methods, additional references, Nature Research reporting summaries, source data, extended data, supplementary information, acknowledgements, peer review information; details of author contributions and competing interests; and statements of data and code availability are available at 10.1038/s41586-022-04688-z.

### Supplementary information

Supplementary Information Supplementary text, figures, equations and references.

### Source data

Source Data Fig. 2

Source Data Fig. 3

Source Data Fig. 4

## Figures and Tables

**Extended Data Table 1:**
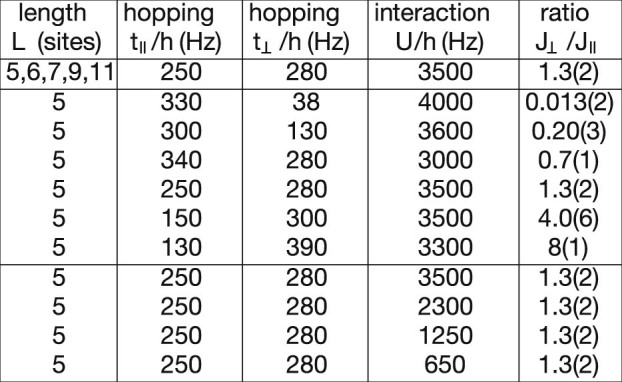
Experimental parameters

The parameters system size *L*, leg coupling ${t}_{\parallel }$, rung coupling ${t}_{\perp }$, interaction *U* and the resulting ratio ${J}_{\perp }/{J}_{\parallel }$ are shown for all datasets. The uncertainties are given for ${J}_{\perp }/{J}_{\parallel }$ and originate from a 5% uncertainty on the hopping parameters ${t}_{\perp }$ and ${t}_{\parallel }$. For the length scan we keep all other parameters constant, whereas the ${J}_{\perp }/{J}_{\parallel }$ scan demands a tuning of both tunnelling amplitudes to keep both $U/{t}_{\parallel }$ and $U/{t}_{\perp }$ high. Where the topologically trivial geometry is realized, it has the same parameters as the topological geometry.

## Data Availability

The datasets generated and analysed during the current study are available from the corresponding author on reasonable request. Source data are provided with this paper.

## Code availability

The code used for the analysis are available from the corresponding author on reasonable request.
