# Supplementary material for: Realizing the symmetry-protected Haldane phase in Fermi–Hubbard ladders
Source: Nature. 2022 Jun 1;606(7914):484–8. doi: 10.1038/s41586-022-04688-z (PMC9200636; doi:10.1038/s41586-022-04688-z)
Supplement: Supplementary file 1 — Supplementary text, figures, equations and references. [file 41586_2022_4688_MOESM1_ESM.pdf]

nature portfolio

https://doi.org/10.1038/s41586-022-04688-z

Supplementary information

Realizing the symmetry-protected Haldane phase in Fermi–Hubbard ladders

In the format provided by the
authors and unedited

Supplementary Information for: Realising the Symmetry-Protected Haldane Phase in Fermi-Hubbard Ladders

Pimonpan Sompet, Sarah Hirthe, Dominik Bourgund, Thomas Chalopin, Julian Bibo, Joannis Koepsell, Petar Bojović, Ruben Verresen, Frank Pollmann, Guillaume Salomon, Christian Gross, Timon A. Hilker, and Immanuel Bloch

In this supplement, we first give additional analyses of bulk and edge observables. Then we discuss the role of the unit cells and present further data on a realization of the Haldane phase with ferromagnetic rung couplings. We finally provide details of the theoretical description of SPT states and derive a string order parameter for the Hubbard chain with density fluctuations.

Triplet fraction in the unit-cell

We investigate the quality of our mapping of the trivial configuration to a spin-0 chain and of the topological configuration to a spin-1 chain (see Fig. 1 of the main text). Numerical results from ED calculations on a system of length $L = 5$ at zero temperature with $M^z = 0$ are shown in Fig. S1. As expected, the rung singlet fraction increases monotonically with $J_\perp/J_\parallel$ and approaches 1 for $J_\perp \gg J_\parallel$. Remarkably the triplet fraction along the diagonals is always high ($\geq 80\%$) and reaches its maximum at $J_\perp/J_\parallel \sim 1$ consistent with [1]. The order parameters in the main text prove that the system stays within the respective trivial (topological) phase even when tuning $J_\perp/J_\parallel$ away from the limiting cases.

Normalisation effects

The string-only correlator $g_{\mathbb{1},R^z}$ is naturally normalised as it returns 1 for any state with an even number of $|S^z| = 1$ in the string. The spin-string correlator g_{S^z,R^z} only equals 1 in a classical spin-1 Néel state. Any spin-1 state with rotational symmetry has $g_{S^z,R^z} < 1$ due to the presence of some $S^z = 0$ at the end of the string. A meaningful normalisation is given by $\tilde{g}_{S^z,R^z} = \eta\, g_{S^z,R^z}$ with $\eta^{-1} = \left\langle \left|\hat{S}^z_k\right| \left|\hat{S}^z_{k+d}\right| \right\rangle$ describing the probability that neither endpoint of the string has spin $S^z_k = 0$. In direct analogy to Bayesian conditional probabilities, $\tilde{g}_{S^z,R^z}$ describes the string-correlation between $|S^z| = 1$ spins and thus $|\tilde{g}_{S^z,R^z}| = 1$ for the AKLT state.

The application of this normalisation is shown using the data of Fig. 2 in Fig. S2a and the explicit values for η are given in Fig. S2b. For $d \gg 1$, it is equivalent to the normalisation given in [1]. We use the same normalisation in the Hubbard regime (see Fig. 4 of the main text).

Finite system size and temperature

In the thermodynamic limit, the SPT phase only exists at strictly zero temperature (i.e. the spin-string correlator $g_{S^z,R^z}(d \to \infty) \neq 0$). The reason is that there are only four ground states, but infinitely many excited states just above the energy gap resulting in infinitely many singlets that can be broken. In our experimental setup, we can nevertheless observe the characteristics of this phase. The finite length of our system limits the number of low energy states available, such that even at a temperature around the gap energy, the ground state is

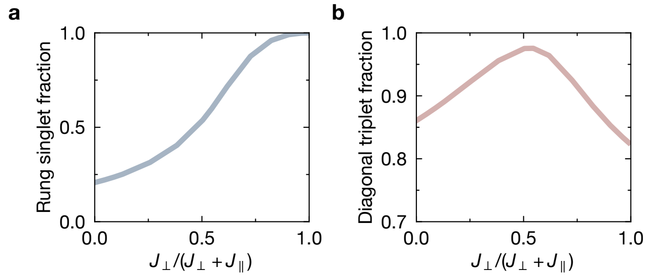

Figure S1. **Singlet and triplet fractions.** **a**, Numerical singlet fraction on the rung of the ladders for different $J_\perp/J_\parallel$. The singlet fraction increases monotonically with the rung coupling. **b**, The fraction of triplets in the diagonal unit-cell is higher than 80% for all $J_\perp/J_\parallel$ and peaks close to 1 when rung and leg coupling become comparable at $J_\perp/J_\parallel \sim 1$. Both plots are calculated on ladders with tilted edges, $L = 5$, $M^z = 0$, and $T = 0$.

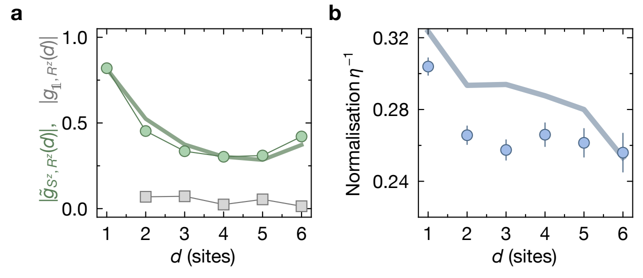

Figure S2. **Normalisation of string correlators.** **a**, Normalised correlator $\tilde{g}_{S^z,R^z}$ compared to $g_{\mathbb{1},R^z}$ in the topological regime measured at length $L = 7, J_\perp/J_\parallel = 1.3(2)$ with $M^z = 0$ and **b**, corresponding values for η^{-1}. Shaded lines are ED calculations at $S/N = 0.45\,k_\mathrm{B}$, $J_\perp/J_\parallel = 1.2$, $L = 7$, $M^z = 0$. At small distances d the normalisation is higher due to correlation between the unit-cells.

still largely populated (see Fig. S3). The colder the system, the longer the length at which the ground state still dominates. Fig. S3a shows the ground state population for short system lengths at $J_\perp/J_\parallel = 1.2$ for $S/N = 0.3\,k_\mathrm{B}$ and $S/N = 0.45\,k_\mathrm{B}$ corresponding to a temperature of $T = 0.6\,J_\parallel,\ 1.2\,J_\parallel$. The ground state population quickly drops as the number of available states increases. The effect of the reduced ground state occupation can be seen in the measured string correlator $g_{S^z,R^z}(d)$ in systems of different length (see Fig. S3b). The system of $L = 11$ shows a much lower value for the string correlator even for short distances, where smaller systems show significantly higher correlations. This restricts the system size up to which signatures of the Haldane phase can be detected in an experiment but even below this system size, all qualitative features of the zero-temperature phase are already present. Furthermore, to show that the signal is not dominated by the small system size, we use DMRG calculations at infinite length at $T = 0.9J_\parallel$, $J_\perp/J_\parallel = 1.3$ (shaded line in Fig. S3b).

Finite size offset

In a finite-size system with fixed total magnetisation, the string correlator g_{S^z,R^z} does not approach zero even when the temperature increases to infinity. The lack of free fluctuations of M^z introduces correlations in the system even for a random distribution of the spins [2]. These correlations, which can be derived from combinatorial considerations, do not depend on the edge termination or coupling parameters. Fig. S4 investigates the effect of this offset on our measurements. As can be seen in Fig. S4a,b, the offset mostly takes sizeable values at the shortest and longest distances of the system. But even at these points, our signal clearly exceeds the offset. Fig. S4c shows the string correlator as a function of the coupling strength (as in Fig. 3a). Interestingly the string correlation value can coincide with or even be lower than the infinite temperature offset for couplings far away from the symmetric point of $J_\perp \approx J_\parallel$. This is however not an artefact of the measurement but is also reflected in the zero temperature ED calculations (shaded line). Thus it is not meaningful to simply subtract this offset. Similarly we show the offset for the Hubbard regime in Fig. S4d. As noted before, the offset is considerably smaller for intermediate string lengths. Therefore we investigate the value of the string correlator for $d = L-2$ (see Fig. S4e). In this case, the offset is considerably smaller than the measured values for any coupling on the topological side. Therefore our system is not dominated by its finite system size but exhibits the topological properties of the bulk characteristic to the Haldane phase.

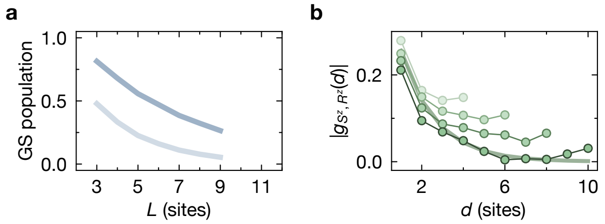

Figure S3. **Finite temperature and finite length effects.** **a**, Numerical ground state (GS) population for a fixed entropy of $S/N = 0.3\,k_\mathrm{B}$ $(0.45\,k_\mathrm{B})$ in (light) blue. The ground state population decreases with length for all finite temperatures. **b**, Experimentally measured string correlator $g_{S^z,R^z}(d)$ for system sizes $L = 5, 7, 9$ and 11 with $J_\perp/J_\parallel = 1.3(2)$. The shaded line shows finite temperature, infinite length DMRG calculations at $J_\perp/J_\parallel = 1.3$, $T = 0.9J_\parallel$.

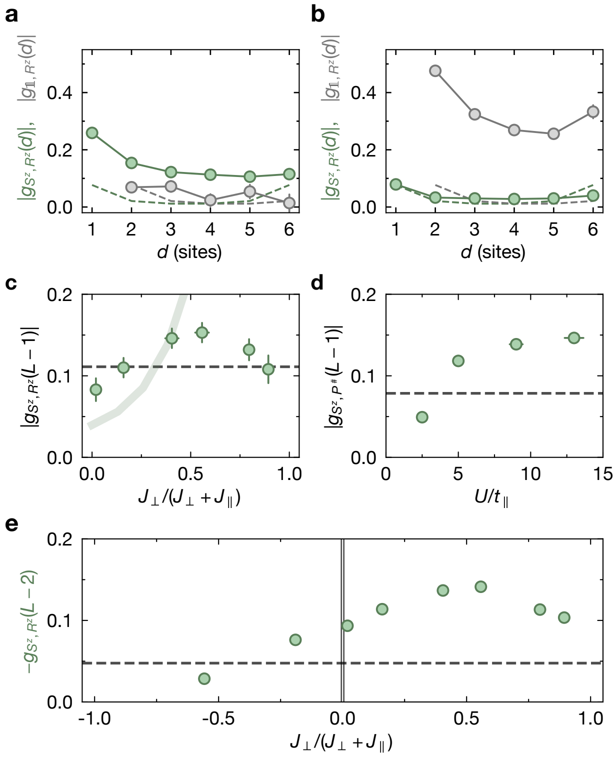

Figure S4. **Finite size offset of the string correlator.** Finite size offset calculated as a function of string distance for the topological (**a**) and trivial (**b**) regime. Dashed lines are numerical calculations, solid lines are measured data presented also in Fig. 2. All values are for $L = 7$, $M^z = 0$ and $J_\perp/J_\parallel = 1.3(2)$. **c**, The finite size offset of the spin string correlator for the infinite temperature Heisenberg model (dashed line) of a topological system with $L = 5$, $M^z = 0$ for different $J_\perp/J_\parallel$. The shaded line shows the zero temperature value from ED calculations. **d**, Similarly, the finite size offset for the Hubbard model compared to the novel spin string correlator (cf. Fig. 4). **e**, String correlator g_{S^z,R^z} at distance $d = L-2$. For all coupling parameters $J_\perp/J_\parallel$ in the topological regime, the measured value clearly exceeds the infinite temperature finite size offset (dashed line).

Microscopy of edge states

Due to our microscopic resolution, we can study the magnetisation pattern within the localised edge state in detail. In Fig. S5, the spatial magnetisation distribution of a system with strong rung coupling is compared to the balanced situation ($J_\perp \approx J_\parallel$). As the $M^z = 1$ sector has positively polarised edge states at $T = 0$, the magnetisation maps directly reveal the structure of the state: the excess magnetisation dominantly sits at the edge but leaks into the bulk where it induces a staggered magnetisation pattern close to the edge due to the AFM spin coupling. We expect the distance that the correlation extends into the bulk to be set by the leg coupling $J_\parallel$ relative to the bulk gap. At finite temperature, there is, in addition, some homogeneous magnetisation of the bulk. In Fig. 3 of the main text, we show the unit-cell average of this data.

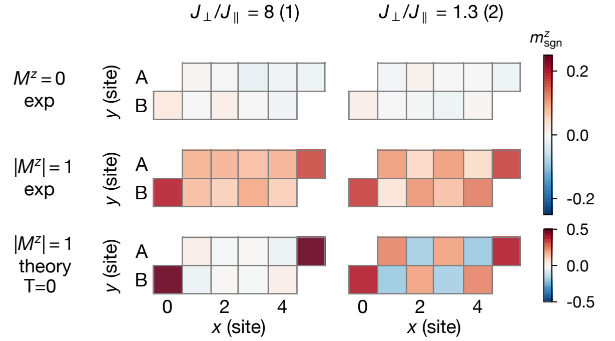

Figure S5. **Spatial magnetisation distribution in the spin-1/2 ladder.** Experimental magnetisation maps $m^z_{x,y}$ for different $J_\perp/J_\parallel$ are shown for $M^z = 0$ in the first row, $|M^z| = 1$ in the second row and zero temperature Heisenberg model at $|M^z| = 1$ in the last row. The plotted quantity is $m^z_{\rm sgn} = m^z_{x,y} \cdot M^z$, flipping the sign in the $M^z = -1$ sector.

Edge state splitting

For finite system length, the four ground states of the SPT phase are not truly degenerate but exhibit a finite energy splitting δ. This energy splitting arises from an exponentially suppressed but non-zero overlap between the edge states, coupling them into singlet and triplet states. Whether the singlet state or the triplet state is lower in energy depends on the parity of the system length L, whereas the energy splitting depends on the system length directly (see Fig. S6a).

Experimentally we cannot observe the splitting directly due to our finite temperature but we explore the underlying spin correlations responsible for the energy splitting: we investigate the effect of even and odd system length by comparing a system of $L = 6$ (see Fig. S6b, c) to one with $L = 5$ (see Fig. S5). The staggered magnetisation pattern seen for $L = 5$ is not visible for $L = 6$ because the induced spin pattern of both edges are incommensurate with each other leading to the higher energy of the triplet state. The opposite is true in the $M^z = 0$ sector, where we find stronger alternating patterns in the even ladder length compared to the odd one (see Fig. S6c around $d = 3, 4$). Here we analyse the spin-correlations $C(1, d) = 4\langle \hat{S}^z_{i,j} \hat{S}^z_{i+1,j+d} \rangle$ because for $M^z = 0$ the local magnetisation is zero everywhere. These observations illustrate how the alternation between singlet and triplet ground states with L is linked to the AFM polarisation due to the edges.

Localisation length

We next relate the experimentally extracted edge state decay length ξ to the localisation of the edge at zero temperature. Numerically, the length over which the edge modes delocalise can be readily extracted from the aforementioned energy splitting δ

$$|\delta| \sim e^{-L/\xi}.$$

The experimental edge decay length is determined, however, using the staggered magnetisation that arises for $|M^z| = 1$

$$|m^z(k)| \sim e^{-k/\xi},$$

where k denotes the position of the unit cell along the chain.

These two approaches are numerically compared in Fig. S7a using DMRG in a system with $L = 100$, $U/t_\parallel = 13$ at zero temperature. The decay length is evaluated from the energy splitting in the different spin sectors $M^z \in \{0, 1\}$. There, we choose $L \in [4, 8, ..., 52]$ and a bond dimension $\chi = 1000$ keeping the maximal energy truncation error below 10^{-7}. For the edge magnetisation, we calculated the ground state in the sector $M^z = 1$ for $L = 100$ and a bond dimension $\chi = 1000$ again with an error below 10^{-7} for all parameters. Both quantities agree with deviations of less than 6% of their values, confirming the validity of our method to extract the localisation length from the experimental data (see Fig. 3). We furthermore compute the bulk correlation length, which is a direct measure of the bulk gap. It can easily be obtained from the ground state of the infinite system [3] and coincides with the former length scales for most parameters.

However, the decay length obtained from the staggered magnetisation shows a strong temperature dependence (see Fig. S7b), whereas the edge state splitting is a property of the spectrum, and thus independent of temperature. The staggered magnetisation arises from the antiferromagnetic correlation of the spin, which decreases with temperature. We find that the edge state cannot delocalise beyond the thermal coherence length of the

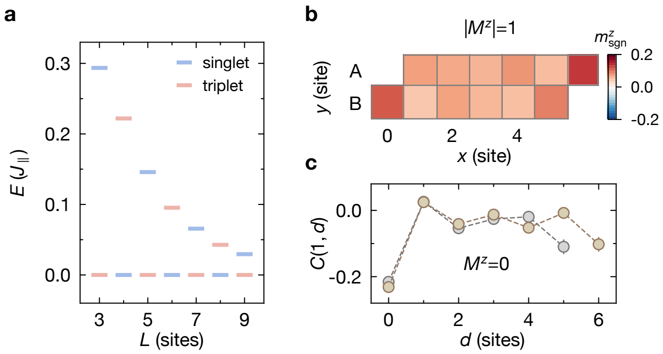

Figure S6. **Edge states for even and odd system length.** **a**, Ground-state energies of the Haldane phase for different system lengths. The lowest-lying state alternates between the singlet and triplet state, depending on the parity of the system length. The data is produced via exact diagonalisation of the spin-1/2-Heisenberg ladder. **b**, The magnetisation map of our system at total magnetisation $|M^z| = 1$ at $L = 6$ and $J_\perp/J_\parallel = 1.3(2)$. It shows the onset of an alternating magnetisation pattern close to the edge sites, but gets lost in the centre of the ladder since the patterns of the two edges have opposite phase. The plotted value m^z_{sgn} is the same as in Fig. S5. **c**, Spin correlations $C(1, d)$ for a system at total magnetisation $M^z = 0$ of $L = 6$ (brown) and of $L = 5$ (grey). Both show a strong nearest-neighbour correlation as well as a strong edge to edge correlation, indicating the edge states of opposite spin for this magnetisation. However, for $L = 6$ the correlations alternate around a finite-size finite-temperature offset, whereas for $L = 5$ they do not alternate because an alternation does not match the length and the negative endpoints.

system. The finite temperature decay length thus follows the zero temperature decay length when it is small (large $J_\perp/J_\parallel$) but saturates in the low $J_\perp/J_\parallel$ regime at an upper bound given by the temperature of the system.

Unit cell and edge effects

In the main text we present two different edge terminations as realisations of the topological and trivial phases. The influence of the edges does however not fully determine the behaviour of the two string correlators. This is to be expected as the string correlators probe the bulk of the system which one could also calculate in an infinite system. Only in conjunction with a chosen unit cell does it make sense to characterise the system via a specific phase.

As an illustrative example we use a singlet chain (see Fig. S8), where every spin-1/2 particle is paired into a singlet with a fixed neighbour. In the case of periodic boundary conditions (a, b, e), the topology of the system with a two-site unit cell is only set by the choice of unit cell: Unit cells around the singlets result in a trivial bulk, while the shifted unit cell leads to a topological Haldane phase. We stress that on the level of spin-1/2

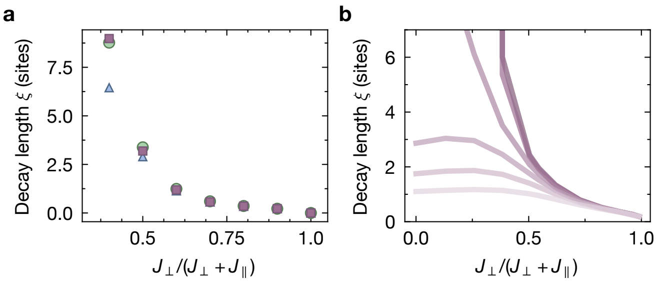

Figure S7. **Decay length comparison.** **a**, The decay length of several quantities is determined using DMRG for a system of $L > 50$, and $U/t_\parallel = 13$. The purple squares show the decay length of the magnetisation pattern, as it is also evaluated on the experimental data. The green circles show the edge localisation length derived from the edge state splitting and the blue triangles show the bulk correlation length. When markers are not visible they coincide with the purple squares. **b**, Finite temperature results for a system with $L = 5$ and $S/N = (0 - 0.5)\,k_{\mathrm{B}}$ (from dark to light purple). The decay length is calculated from the magnetisation pattern in the Heisenberg model using ED.

particles both states are identical and only the different choices of pairing in the analysis lead to the different topologies.

By cutting, the ring can be turned into a chain with edges. But because there is no long-range entanglement in the system, the bulk properties stay unaffected by the cut i.e. the topology is still set by the choice of unit cell and not by the position of the cut. Only one of the two choices of units cell, however, agrees with the cut such that no unit cell is split (c, g). By providing such a natural choice of unit cell, the edge is linked to the topology of the bulk. When only considering the bulk it is also possible to choose the opposite unit cell, which disagrees with the cut, leading to the opposite string-correlation results (d, f).

In the main text, we presented results for the natural choice of unit cells (vertical for straight edges, diagonal for tilted edges). In Fig. S8h,i, we compare the string correlators g_{S^z,R^z} and $g_{\mathbb{1},R^z}$ with both edge terminations at fixed unit cells. In tilted unit cells (h), we always find a topological bulk, while straight unit cells (i) show the trivial correlations. The physical presence or absence of an unpaired spin-1/2 does not affect the bulk significantly. This is consistent with the discussion in the last paragraph and demonstrates, that, even in our relatively small systems, the properties of the bulk are independent of the edge.

An additional method to realise the Haldane phase

As just explained, the identification of the topological and trivial phases in our main experimental dataset

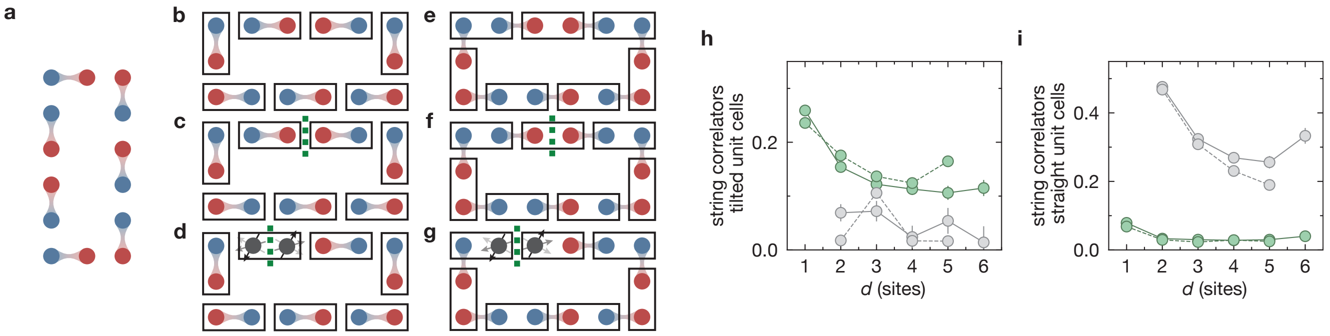

Figure S8. **Illustration of the relationship between edges and unit cells.** **a**, Ring of singlets without any predefined unit cell. No clear statement about the topological or trivial phases can be made here. On the right, the first column (**b**, **c**, **d**) uses unit cells aligned with the singlet bonds (which corresponds to vertical unit cells in the experiment). The second column (**e**, **f**, **g**) has unit cells connecting different singlets, i.e. diagonal unit cells. **b** (**e**) Periodic boundary conditions with vertical (diagonal) unit cells. The system is in the trivial (topological) phase, but shows no edge states. Panel **c** and **f** cut the system into a chain while leaving the singlets intact. This corresponds to the system with straight edges. There is no spin-1/2 edge state. Meanwhile **d** and **g** cut singlets as in the system with tilted edges and produce a free spin-1/2 at the system edge. Note that only in **c** and **g** the definition of the unit cell agrees with the cut of the system leading to the terminology of a trivial chain for straight edges and topological chain for tilted edges. **h** [**i**] shows the string correlators g_{S^z,R^z} (green) and $g_{\mathbb{1},R^z}$ (grey) for diagonal [vertical] unit cells. Solid lines correspond to edges matching the unit cell (i.e. tilted edges for the diagonal unit cell and vice versa), dashed lines to opposite orientation such that there are half unit cells at the edges. The choice of edge termination only has a minor effect on the signal.

is based on the fact that a system with diagonal unit cells can be effectively described by a Heisenberg spin-1 chain, while the vertical unit-cell ladders are described by a spin-0 chain. Another way to realise the Haldane phase is to (actively) change the bulk couplings instead of (passively) changing the unit cell. For ferromagnetic (FM) couplings along the rungs of the ladders, $J_\perp < 0$, the vertical unit cell carries a spin $S = 1$ object, such that a straight edge system is effectively described by a Heisenberg spin-1 chain.

We experimentally realise such rung-FM ladders by applying a local potential offset Δ on one of the legs of the ladders (see Fig. S9b), using the light-shift generated by the DMD. This allows to tune the superexchange coupling $J_\perp$ [4, 5] ; in the regime $\Delta > U$, the sign of the superexchange is reversed, leading to FM rung coupling $J_\perp < 0$. For our parameters, we estimate the strength of the coupling to $J_\perp/(|J_\perp| + J_\parallel) = -0.55(13)$, and local ferromagnetism is confirmed by evaluating the nearest neighbour spin correlations of the underlying spin-1/2 system, $C_y = 4\langle \hat{S}^z_{A,j}\hat{S}^z_{B,j}\rangle \approx 0.1$, i.e. 30% of the maximal value in a SU(2)-symmetric system.

We show in Fig. S9 the string correlators g_{S^z,R^z} and $g_{\mathbb{1},R^z}$ as a function of distance, as well as the magnetised pattern along a chain in the sector of total magnetisation $|M^z| = 1$. We observe the same signatures of the Haldane phase as the ones presented in the main text (Fig. 2). Hidden antiferromagnetic order is revealed by the string-spin correlator $g_{S^z,R^z}(d)$ (Fig. S9c) which remains nonzero across the whole system length, while the two-point spin-1 correlator $C(d)$ rapidly decays to zero (inset). The string-only correlator $g_{\mathbb{1},R^z}(d)$ is smaller than g_{S^z,R^z} in the relevant range of large d.

In Fig. S9d, we show the decaying magnetisation pattern that emerges in the sector of total magnetisation $|M^z| = 1$, revealing the presence of edge states. The extracted decay length is $\xi = 1.3(1)$, similar to the decay length in the AFM-ladders with tilted edges (Fig. 3).

Symmetry fractionalisation

The key to understanding one-dimensional SPT phases is the notion of symmetry fractionalisation [6, 7]. If $\hat{U} = \prod_n \hat{U}_n$ is an on-site unitary symmetry, it can be argued that if the ground state $|\psi\rangle$ is symmetric under $\hat{U}$ and has a finite correlation length, then

$$\prod_{k=m}^{n} \hat{U}_k|\psi\rangle = \hat{U}_L\hat{U}_R|\psi\rangle, \tag{1}$$

where $\hat{U}_L$ ($\hat{U}_R$) is a unitary operator which is exponentially localised to the left (right) of the block of sites $k = m, m+1, \cdots, n-1, n$. Moreover, if $\hat{V}$ is another symmetry, then the group relations between the fractionalised symmetries $\hat{V}_L$ and $\hat{U}_L$ are the same as those between the bulk symmetries $\hat{V}$ and $\hat{U}$ up to potential phase factors. As an example, suppose that $\hat{U}$ and $\hat{V}$ commute and suppose that $\hat{U}_{L,R}$ are bosonic operators (which is the case for all symmetries considered in this work), then

$$\mathbb{1} = (\hat{U}_L\hat{U}_R)(\hat{V}_L\hat{V}_R)(\hat{U}_L\hat{U}_R)^{-1}(\hat{V}_L\hat{V}_R)^{-1} \tag{2}$$

$$= \left(\hat{U}_L\hat{V}_L\hat{U}_L^{-1}\hat{V}_L^{-1}\right)\left(\hat{U}_R\hat{V}_R\hat{U}_R^{-1}\hat{V}_R^{-1}\right). \tag{3}$$

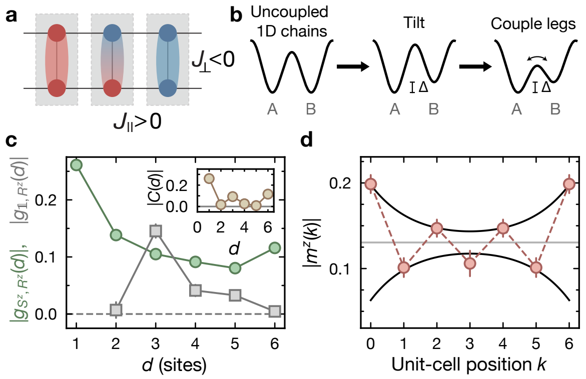

Figure S9. **Signatures of the Haldane phase in ladders with ferromagnetic rung coupling.** **a** In rung-FM ladders ($J_\perp < 0$), the vertical unit cell carries a $S = 1$ object. **b** We engineer such ladders by applying a potential offset $\Delta > U$ on one of the legs of the ladders. The experimental preparation is designed to prevent atoms from tunneling from leg A to leg B while applying the tilt. **c** The string-spin g_{S^z,R^z} and string-only $g_{\mathbb{1},R^z}$ correlators are evaluated as a function of the rung distance d in straight-edge ladders (vertical unit cells) of length $L = 7$ with FM coupling along the rungs, in the sector of fixed total magnetisation $M^z = 0$. Their behavior is analogous to the case of AFM ladders with tilted edges (diagonal unit cell, Fig. 2b of the main text), consistent with symmetry-protected topological order. (Inset) The two-point spin-spin correlator $C(d)$ decays rapidly to zero as a function of the distance d. **d**. In the magnetised sector $|M^z| = 1$ of the rung-FM ladder, the (vertical) unit-cell averaged magnetization $|m_z(k)|$ shows a staggered pattern with decaying amplitude, similar to the one observed in the AFM ladder with tilted edges (Fig. 3b), indicating the presence of edge states.

Since the fractionalised operators on the left and right have disjoint support (up to exponentially small overlaps), we have that $\hat{U}_L \hat{V}_L \hat{U}_L^{-1} \hat{V}_L^{-1} = e^{i\alpha}\mathbb{1}$, i.e., $\hat{U}_L \hat{V}_L = e^{i\alpha} V_L \hat{U}_L$: the fractionalised symmetries commute up to a phase. More generally, the group relations of the fractionalised symmetries define a *projective representation* of the symmetry group. Some of these phase factors can be gauged away by redefining $\hat{U}_L, \hat{U}_R \to e^{i\beta}\hat{U}_L, e^{-i\beta}\hat{U}_R$, while other phase factors are invariant. The collection of such invariant phase factors define the so-called second group cohomology class $H^2(G, U(1))$: any non-zero element in this class represents a non-trivial SPT phase. The Haldane SPT case corresponds to where the bulk symmetry group is $SO(3)$ (or its $\mathbb{Z}_2 \times \mathbb{Z}_2$ subgroup) and the fractionalised symmetries form $SU(2)$ (or its quaternion subgroup).

From symmetry fractionalisation to edge modes

Note that a non-trivial projective representation for the fractionalised symmetries automatically implies edge modes: for open boundaries, one can consider Eq. (1) as acting on the whole system, which moreover implies that $\hat{U}_L$ and $\hat{U}_R$ are genuine symmetries of the ground state. Since a projective representation can never act on a one-dimensional Hilbert space, there must be a degenerate zero-energy Hilbert space associated to the boundaries (in other words, the ground state cannot be a simultaneous eigenstate of all fractionalised symmetries).

From symmetry fractionalisation to string order parameters

For an on-site symmetry $\hat{U} = \prod_n \hat{U}_n$, consider a string operator $\hat{\mathcal{O}}_m^\dagger \hat{U}_{m+1} \cdots \hat{U}_{n-1} \hat{\mathcal{O}}_n$ with endpoint operator $\hat{\mathcal{O}}_n$ and $n - m$ being much larger than the correlation length. Using symmetry fractionalisation, its expectation value can be expressed as

$$\langle \hat{\mathcal{O}}_m^\dagger \hat{U}_{m+1} \cdots \hat{U}_{n-1} \hat{\mathcal{O}}_n \rangle = \langle \hat{\mathcal{O}}^\dagger \hat{U}_L \rangle \langle \hat{U}_R \hat{\mathcal{O}} \rangle. \qquad (4)$$

Hence, long-range order (LRO) for this string operator is equivalent to $\langle \hat{\mathcal{O}}^\dagger \hat{U}_L \rangle \neq 0$. In the assumption that the ground state is symmetric (i.e., no spontaneous symmetry breaking), a local operator can only have a non-zero expectation value if it is neutral under the symmetry group. This means that LRO is only possible if $\hat{\mathcal{O}}$ is chosen to have the same symmetry charges as the fractionalised symmetry $\hat{U}_L$ (i.e., finding the right $\hat{\mathcal{O}}$ allows to infer the projective representation of the fractionalised symmetries). This is how string order parameters can be used to diagnose an SPT phase [8].

As an example, consider an on-site $\mathbb{Z}_2 \times \mathbb{Z}_2$ symmetry, such as the π-rotations $\hat{R}_x$ and $\hat{R}_z$ for an integer spin chain. The fractionalised symmetries either commute or anticommute: $\hat{R}_x^L \hat{R}_z^L = \pm \hat{R}_z^L \hat{R}_x^L$; the anticommuting case gives rise to the topological Haldane phase. In particular, this means that $\hat{R}_x \hat{R}_z^L \hat{R}_x^\dagger = \hat{R}_x^L \hat{R}_z^L \left(\hat{R}_x^L\right)^\dagger = -\hat{R}_z^L$. Hence, a string for $\hat{R}_z$ can only have LRO if the endpoint operator is odd under $\hat{R}_x$. This explains the well-known string order parameter for the Haldane SPT phase: $\cdots \hat{R}_z \hat{R}_z \hat{R}_z \hat{S}^z$. Conversely, the trivial phase can only have LRO for the $\hat{R}_z$ string if the endpoint is even under $\hat{R}_x$; choosing the endpoint operator to be the identity operator does the trick.

If one tunes away from the Mott limit, the distinction between these two cases breaks down. The integer spin chain now does not have $SO(3)$ symmetry but rather $SU(2)$ symmetry. More concretely, $\hat{R}_x$ and $\hat{R}_z$ are no longer $\mathbb{Z}_2$ symmetries: they square to fermion parity symmetry $\hat{P}$, not to the identity. (In the Mott limit, fermion parity is a classical number in each unit cell, giving rise to an effective spin chain.) This means that the fractionalised symmetries now obey $\hat{R}_x^L \hat{R}_z^L = \hat{P}^L \hat{R}_z^L \hat{R}_x^L$, which allows to adiabatically connect the spin chains where $\hat{P}^L = \pm\mathbb{1}$ as has been demonstrated before [9–11].

A novel string order parameter for anti-unitary symmetry

Let $\hat{T}$ be an anti-unitary symmetry. Analogous to Eq. (1), its symmetry fractionalisation can be written as $\hat{T} = \hat{U}_L \hat{U}_R \tilde{K}$, where $\tilde{K}$ is complex conjugation defined with respect to a basis that factorises between left and right (see [6, 12] for details). A single anti-unitary $\mathbb{Z}_2^T$ symmetry can protect a non-trivial SPT phase. More precisely, $\hat{T}^2$ implies that $\hat{U}_L \tilde{K} \hat{U}_L \tilde{K} = \hat{U}_R \tilde{K} \hat{U}_R \tilde{K} = e^{i\theta} = \pm 1$; the case $\theta = \pi$ is the topological Haldane phase, where the edge mode is a Kramers pair under $\hat{T}$.

Usually, it is said that there is no simple string order parameter to detect such an SPT phase protected by $\mathbb{Z}_2^T$. The simplest "string order" is rather involved, requiring two copies of the system and a partial swap [8]. Here, we show that a conventional string order parameter can be constructed if the system has an *additional* unitary $\mathbb{Z}_2$ symmetry, which we denote as $\hat{P}$.

Suppose that we consider phases which are not protected by the combined symmetry $\hat{P}\hat{T}$. We now show that the string order parameter for a $\hat{P}$-string, i.e., $\hat{\mathcal{O}}_m^\dagger \hat{P}_{m+1} \cdots \hat{P}_{n-1} \hat{\mathcal{O}}_n$, can be used to diagnose whether the phase is in a trivial or topological phase with respect to the anti-unitary symmetry $\hat{T}$. To see this, first note that the phase being trivial with respect to $\hat{P}\hat{T}$ implies that if $\hat{P} = \hat{P}_L \hat{P}_R$ is the fractionalisation of $\hat{P}$, then $\hat{P}\hat{T} = \hat{P}_L \hat{U}_L \hat{P}_R \hat{U}_R \tilde{K}$ must obey $\hat{P}_L \hat{U}_L \tilde{K} \hat{P}_L \hat{U}_L \tilde{K} = +1$. Moreover, since $\hat{P}^2 = 1$, we can choose $\hat{P}_L^2 = 1$, such that

$$\hat{P}_L = \hat{U}_L \tilde{K} \hat{P}_L \hat{U}_L \tilde{K}. \tag{5}$$

We thus obtain $\hat{T}\hat{P}_L\hat{T} = \hat{U}_L \hat{U}_R \tilde{K} \hat{P}_L \hat{U}_L \hat{U}_R \tilde{K} = \left(\hat{U}_L \tilde{K} \hat{P}_L \hat{U}_L \tilde{K}\right)\left(\hat{U}_R \tilde{K} \hat{U}_R \tilde{K}\right) = e^{i\theta} \hat{P}_L$. Hence, whether $\hat{P}_L$ commutes or anticommutes with $\hat{T}$ encodes what phase we are in. Using the reasoning of the previous section, one can conclude that the string order parameter for $\hat{P}$ with endpoint operator $\hat{\mathcal{O}}$ (where we choose $\hat{\mathcal{O}}$ to be hermitian) has long-range order if $\hat{T}\hat{\mathcal{O}}_n\hat{T} = e^{i\theta}\hat{\mathcal{O}}_n$.

Application to Hubbard chain

We have already explained why away from the Mott limit, we can no longer rely on the conventional string order parameter to characterise an SPT phase. However, as pointed out in [12], the (bond-alternating) Hubbard chain is still in a non-trivial SPT phase protected by an anti-unitary $\mathbb{Z}_2^T$ symmetry. This uses the bipartite structure of our model (which is evident in the fact that the system lives on a ladder), leading to the symmetry as defined by

$$\hat{T} : c_{x,y,s} \leftrightarrow (-1)^{x+y} c_{x,y,s}^\dagger \tag{6}$$

where $x = [0, L]$, $y = 0, 1$ (corresponding to A or B) and $s = \uparrow, \downarrow$. Here the bipartite property is encoded in the factor $(-1)^{x+y}$ as the ladder Hamiltonian only couples sites with opposite parity. This follows directly from combining two facts: (i) it is a $\mathbb{Z}_2^T$ symmetry of the model, even away from the Mott limit, and (ii) in the Mott limit, it can be argued that it coincides with spinful time-reversal symmetry [12], which is known to protect the Haldane SPT phase [6]. Experimentally this sublattice symmetry is realised very well with same-sublattice couplings being smaller than 4% of the nearest neighbour tunneling amplitude at $V = 6.3\,E_{\mathrm{R}}$. Compared to the effect of finite temperature on the system, these couplings are negligible and could even be arbitrarily suppressed by increasing the lattice depth. Furthermore any differences in chemical potential between the sublattices are removed by the procedure to generate homogeneous systems which is unaware of the sublattice structure.

To construct a string order parameter for this phase, we use the result obtained in the previous section. In particular, consider the additional $\mathbb{Z}_2$ symmetry $\hat{P}_\downarrow$, which is the fermion parity of the down-spin species. In the Mott limit, this symmetry becomes indistinguishable from $\hat{R}_z$. From this, we learn that $\hat{P}_\downarrow\hat{T}$ does not protect the SPT phase (which was a condition that we assumed in the previous section). As derived above, this implies that the string operator associated to $\hat{P}_\downarrow$ can be used to read off the topological invariant: we are in the Haldane SPT (trivial) phase if the string has long-range order for an endpoint operator that is odd (even) under $\hat{T}$. For instance, for the topological phase, we can thus choose the endpoint operator $\hat{\mathcal{O}}_n = \hat{S}_n^z = \frac{1}{2}\left(\hat{c}_{n,\uparrow}^\dagger \hat{c}_{n,\uparrow} - \hat{c}_{n,\downarrow}^\dagger \hat{c}_{n,\downarrow}\right)$, which is odd under the above anti-unitary $\hat{T}$ symmetry.

[1] White, S. R. Equivalence of the antiferromagnetic Heisenberg ladder to a single S=1 chain. *Phys. Rev. B* **53**, 52 (1996).

[2] Hilker, T. A. *et al.* Revealing hidden antiferromagnetic correlations in doped Hubbard chains via string correlators. *Science* **357**, 484 (2017).

[3] Schollwöck, U. The density-matrix renormalization group in the age of matrix product states. *Ann. Phys.* **326**, 96 (2011).

[4] Duan, L.-M *et al.* Controlling Spin Exchange Interactions of Ultracold Atoms in Optical Lattices *Phys. Rev. Lett.* **91**, 090402 (2003).

[5] Trotzky, S. *et al.* Time-Resolved Observation and Control of Superexchange Interactions with Ultracold Atoms in Optical Lattices *Science* **319**, 295 (2008).

[6] Pollmann, F., Turner, A. M., Berg, E. & Oshikawa, M.

Entanglement spectrum of a topological phase in one dimension. *Phys. Rev. B* **81**, 064439 (2010).

[7] Chen, X., Gu, Z.-C. & Wen, X.-G. Classification of gapped symmetric phases in one-dimensional spin systems. *Phys. Rev. B* **83**, 035107 (2011).

[8] Pollmann, F. & Turner, A. M. Detection of symmetry-protected topological phases in one dimension. *Phys. Rev. B* **86**, 125441 (2012).

[9] Anfuso, F. & Rosch, A. Fragility of string orders. *Phys. Rev. B* **76**, 085124 (2007).

[10] Moudgalya, S. & Pollmann, F. Fragility of symmetry-protected topological order on a Hubbard ladder. *Phys. Rev. B* **91**, 155128 (2015).

[11] Verresen, R., Bibo, J. & Pollmann, F. Quotient symmetry protected topological phenomena. *arXiv:2102.08967* (2021).

[12] Verresen, R., Moessner, R. & Pollmann, F. One-dimensional symmetry protected topological phases and their transitions. *Phys. Rev. B* **96**, 165124 (2017).
